# Protection of Wine from Protein Haze Using *Schizosaccharomyces japonicus* Polysaccharides

**DOI:** 10.3390/foods9101407

**Published:** 2020-10-03

**Authors:** Valentina Millarini, Simone Ignesti, Sara Cappelli, Giovanni Ferraro, Alessandra Adessi, Bruno Zanoni, Emiliano Fratini, Paola Domizio

**Affiliations:** 1 Department of Agriculture, Food, Environment and Forestry (DAGRI)—University of Florence, P.le delle Cascine 18, 50144 Florence, Italy; valentina.millarini@unifi.it (V.M.); simone.ignesti@gmail.com (S.I.); saracappelli11194@gmail.com (S.C.); alessandra.adessi@unifi.it (A.A.); bruno.zanoni@unifi.it (B.Z.); 2 Department of Chemistry “Ugo Schiff” and Center for Colloid and Surface Science (CSGI)—University of Florence, Via della Lastruccia 3-13, 50019 Sesto Fiorentino, Italy; giovanni.ferraro@unifi.it (G.F.); emiliano.fratini@unifi.it (E.F.)

**Keywords:** wine protein, wine haze, protein stability test, protein stability treatment, mannoprotein, polysaccharide, *Schizosaccharomyces japonicus*, non-*Saccharomyces*

## Abstract

Nowadays commercial preparations of yeast polysaccharides (PSs), in particular mannoproteins, are widely used for wine colloidal and tartrate salt stabilization. In this context, the industry has developed different processes for the isolation and purification of PSs from the cell wall of *Saccharomyces cerevisiae*. This yeast releases limited amounts of mannoproteins in the growth medium, thus making their direct isolation from the culture broth not economically feasible. On the contrary, *Schizosaccharomyces japonicus*, a non-*Saccharomyces* yeast isolated from wine, releases significant amounts of PSs during the alcoholic fermentation. In the present work, PSs released by *Sch. japonicus* were recovered from the growth medium by ultrafiltration and their impact on the wine colloidal stability was evaluated. Interestingly, these PSs contribute positively to the wine protein stability. The visible haziness of the heat-treated wine decreases as the concentration of added PSs increases. Gel electrophoresis results of the haze and of the supernatant after the heat stability test are consistent with the turbidity measurements. Moreover, particle size distributions of the heat-treated wines, as obtained by Dynamic Light Scattering (DLS), show a reduction in the average dimension of the protein aggregates as the concentration of added PSs increases.

## 1. Introduction

The use of bentonite represents the most common method used worldwide for wine protein stabilization [1]. Bentonite acts as a cation exchanger and, by binding to proteins present in the wine through electrostatic interactions, forms complexes that can be then removed by filtration. Although this kind of adjuvant is very effective in removing proteins responsible for haze, it has several drawbacks like the removal of color and flavor compounds [2,3,4]. Moreover, the disposal of spent bentonite requires extra labor costs and, in addition, presents health problems associated with its management. 

Due to the negative implications associated with bentonite, several alternatives to its use have been explored such as flash pasteurization [5,6], ultrafiltration [7,8], addition of proteolytic enzymes [9,10] silica gel, hydroxyapatite and alumina [11], zirconium oxide [12,13,14,15], natural zeolites [16,17], chitin and chitosan [18,19,20,21], carrageenan [22,23,24], and yeast mannoproteins [25,26,27,28,29,30]. These last ones have recently found an increasing interest in the wine industry as a result of the multiple positive effects associated with this bio-product: decreasing astringency [31], improving mouth-feel and fullness [32], adding complexity and aromatic persistence [33], increasing sweetness and roundness [34], and reducing protein and tartrate instability [35,36].

Mannoproteins are highly glycosylated proteins, containing 1–10% of proteins and 85–90% of carbohydrates, mainly mannose [37,38]. Because of their high content of carbohydrates, mannoproteins are normally referred to as polysaccharides. Mannoproteins are present on the outer cell wall layer of yeast and are released during alcoholic fermentation and wine aging processes [39,40,41,42].

A competition between wine proteins and mannoproteins for non-proteinaceous wine components (responsible for wine protein instability) has been reported as the most likely mechanism to explain the improved wine stability obtained through mannoproteins [43]. 

Although some scientific evidence has already emphasized the positive impact on wine protein stability determined by mannoproteins purified from wine or from yeast cell wall of *S. cerevisiae* [25,26,27,28,29,30], the efficacy of most of the commercial exogenous mannoproteins of *Saccharomyces* is very limited. A different impact on the wine protein stability by eleven commercial mannoprotein preparations was highlighted by Ribeiro et al. [44]. Interestingly, it was found that mannoproteins with a low mannose-to-glucose ratio were not able to stabilize wine against protein instability, highlighting that their performances are strictly related to the different chemical composition and the different degrees of purity, or more practically to the extraction and purification methods adopted by individual manufacturers. 

Various processes for the isolation and purification of mannoproteins can be applied, such as enzymatic treatments (to release them from the cell wall) and acids or hot alkali usage (to solubilize proteins). However, these extraction methods can affect the structural features and molecular weight of these macromolecules and, in turn, can significantly affect their bioactivity [45]. On the other hand, the extraction of mannoproteins from the cell wall of *Saccharomyces* yeasts allows a higher yield as compared to their recovery from the fermentation medium. Indeed, *Saccharomyces* yeasts, during alcoholic fermentation, release a rather low quantity of mannoproteins, normally ranging from 50 to 175 mg/L [29,46]. However, yeasts other than *Saccharomyces* are able to release larger amounts of polysaccharides (PSs) [47,48,49,50,51,52]. In particular, Domizio et al. [51] have shown that yeasts belonging to the species *Schizosaccharomyces japonicus* were able to release a high quantity of polysaccharides in the fermentation medium. In particular, the strains *Sch. japonicus* # UCD2489 released an amount of PSs ~7 times higher than that released by a commercial *Saccharomyces cerevisiae* yeast strain under the same fermentative conditions. Interestingly, Domizio et al. [53], in a study carried out using mixed fermentation of *Sch. japonicus/S. cerevisiae*, found that polysaccharides released by *Sch. japonicus* were able to protect wine from protein haze. 

The possibility of recovering these macromolecules directly from the growth media by ultrafiltration would overcome the necessity of the extraction of PSs from the cell wall, maintaining their native structure unaltered. Based on these observations, the aim of this study was to evaluate the impact of *Sch. japonicus* # UCD2489 polysaccharides on wine protein stability.

## 2. Materials and Methods

### 2.1. Yeast Strains

A yeast strain belonging to the species *Sch. japonicus* (UCD2489) from the yeast culture collection of the Department of Viticulture and Enology University of California, Davis, was used.

### 2.2. Wine

Vernaccia di San Gimignano, a Tuscan white wine, was used in the present study to assess the impact of *Sch. japonicus* polysaccharides on wine protein stability. Table 1 shows the main chemical characteristics of Vernaccia wine, as obtained by Fourier-transform infrared spectroscopy (FT-IR) (FOSS WineScan, FT 120 Reference Manual, Foss, Hamburg, Germany). 

### 2.3. Fermentation Conditions

Fermentations were carried out at 27 °C in 500 mL Erlenmeyer flasks containing 330 mL of a synthetic polysaccharide-free grape juice “Minimal Must Medium” (MMM) [54], with no addition of Tween 80 and ergosterol. The concentrations of assimilable nitrogen and sugar were 208 mg/L and 120 g/L, respectively. The medium was sterilized by filtration. The preculture of *Sch. japonicus* # UCD2489 was grown in 10 mL of the same modified synthetic medium at 27 °C for 72 h, and then used to inoculate the fermentations at the optical density of 0.1 (OD_600_ nm). The flasks, closed with a cotton plug, were continuously agitated at 150 rpm in an orbital shaker. The fermentation kinetic was monitored by weight loss, due to CO_2_ production, and was followed for ten days.

### 2.4. Polysaccharides Recovery and Purification

After ten days of alcoholic fermentation, cells were removed by centrifugation (8000 g, 4 °C, 15 min) and the supernatant was filtered through 0.45 μm acetate cellulose membranes. Afterwards, the filtered supernatant was dialyzed and concentrated using an ultrafiltration unit (Amicon^®^-stirred cell 8200, Millipore, Bedford, MA, USA) equipped with a polyethersulphone membrane with a 10 kDa molecular weight cut-off (PBGC06210, Millipore, Bedford, MA, USA). The ultrafiltration was carried out using a N_2_ pressure of around ~30 psi. The retentate was collected and freeze-dried for 48 h (Edwards Modulyo freeze-dryer, Edwards, Crawley, UK). 

### 2.5. Wine Treatments

Increasing concentrations of freeze-dried polysaccharides (UFS) were added into the Vernaccia wine. In particular, 1 g/L of UFS was resuspended in the Vernaccia wine (stock solution) and then diluted (using the same wine) to prepare different aliquots at lower concentrations (100 mg/L; 200 mg/L; 300 mg/L; 400 mg/L and 600 mg/L). Three aliquots of each concentration were prepared. All the operations were performed in a laminar flow hood to prevent microbial and dust contamination.

### 2.6. Polysaccharides Characterization

#### 2.6.1. Polysaccharides Quantification

The concentration of PSs was evaluated by high-performance liquid chromatography (HPLC), according to the method reported in Romani et al. [55]. After filtration through 0.45 μm nitrocellulose membranes, 20 μL of sample were injected into the HPLC apparatus (Varian Inc., Palo Alto, CA, USA) equipped with a 410 series autosampler, a 210 series pump, and a 356-LC refractive index detector. Isocratic separation was performed on a TSK G-OLIGO-PW (808031) column (30 cm × 7.8 mm i.d.) and a TSK-GEL OLIGO (808034) guard column (4 cm × 6 mm i.d.) (Supelco, Bellefonte, PA, USA). The mobile phase was 0.2M NaCl, at a flow rate of 0.8 mL/min. Peaks were quantified by comparison with an external calibration curve of mannan (Sigma-Aldrich, Milan, Italy) from 50 mg/L to 1000 mg/L. The analysis of the peaks was performed using the software Galaxie Chromatography Data System (version 1.9.302.530) (Varian Inc., Palo Alto, CA, USA). All the analyses were carried out in duplicate.

#### 2.6.2. Monosaccharide Composition 

Polysaccharides were first hydrolyzed with trifluoroacetic acid (TFA). In particular, 5 mg of UFS were mixed with 2 mL of a 2 N TFA solution and heated at 120 °C for 120 min. TFA was then removed using a rotary evaporator and the dried extracts were re-solubilized in deionized water. This operation was repeated three times per sample.

The monosaccharidic composition of the hydrolyzed fraction was analyzed using Ion Exchange Chromatography (IEC) following the procedures described by Chamizo et al. [56]. A Dionex ICS-2500 ion exchange chromatograph, equipped with an ED50 pulsed amperometric detector operating with a gold working electrode, was used. Each sample was injected into a Dionex CarboPacPA1 column (4.6 × 250 mm, Thermo Scientific, Waltham, MA, USA). The eluents HPLC-grade water (A), 0.185 M NaOH (B), and 0.488 M sodium acetate (C), were mixed as follows: from injection time to 20 min A:B = 90:10; from 20 to 30 min B:C = 50:50; from 30 to 60 min, A:B = 90:10. The flow rate was kept at a constant value of 1 mL/min. The different sugars were identified on the basis of the retention time of known standards. Results were expressed as molar ratio (%).

#### 2.6.3. Protein Quantification

The freeze-dried polysaccharides (UFS) were rehydrated with a known amount of distilled water for protein quantification. Quantitative determination of proteins was assessed by dye-binding Bradford assay [57] using bovine serum albumin (BSA) (Sigma-Aldrich, Milan, Italy) and dye reagent (Bio-Rad Laboratories, Hercules, CA, USA) for the calibration curve. 

### 2.7. Wine Protein Heat Test and Treatment of the Derived Fractions

Protein stability was assessed by determining the induced haze value following the heat test according to McRae et al. [58]. Briefly, wine aliquots were first filtered (0.45 μm, acetate cellulose membranes) and then heated at 80 °C for 2 h. Successively, these aliquots were cooled at 4 °C for 16 h and left at room temperature for 2 h before measuring the turbidity. Wine turbidity was determined with a nephelometer (HI88703 turbidimeter, Hanna Instrument Inc., Woonsocket, USA). Data were analyzed by One-way ANOVA and Tukey’s post-test, setting *p* value to 0.05.

After the heat test, the wines were centrifuged in order to separate the haze fraction (HF) from the supernatant fraction (SF). Whereupon, SF was added with four volumes of cold 95% ethanol containing HCl 0.3 M and kept at 4 °C for 24 h to precipitate the polysaccharides. After centrifugation (9000× *g*, 4 °C, 30 min), the supernatant was discarded, and the pellet was washed twice with four volumes of 96% *v/v* cold ethanol and finally vacuum-dried at room temperature. The dried pellet was then rehydrated with distilled water for the successive proteins profiling characterization. Instead, HF was added to the sodium dodecyl sulfate sample buffer. 

### 2.8. Proteins Profiling by Gel Electrophoresis

Detection of proteins was performed by using 10% sodium dodecyl sulfate-polyacrylamide gel electrophoresis (SDS-PAGE) [59]. In particular, 20 μL of sample was treated with 6.65 μL of 4X Laemmlli buffer (Bio-Rad) and 2.75 μL of 1 M dithiothreitol (DTT) (Acros Organics, Geel, Belgium ) and then heated at 95 °C for 5 min. Afterwards, 15 μL of the mixture was loaded onto the gel. Blue precision plus protein standard (Bio-Rad) was loaded. The SDS-PAGE was performed using a Mini Protean II apparatus (Bio-Rad) (45 V within the stacking gel and 104 V in the developing gel). The protein bands in the gels were stained with Bio-Safe Coomassie G-250 stain (Bio-Rad). Instead, glycoproteins were detected with the periodic acid Schiff’s reagent (Sigma-Aldrich) applying the procedure described by Packer et al. [60]. Briefly, the SDS gel was fixed in 50% (*v/v*) ethanol for 30 min, washed in distilled water for 10 min and then incubated for 30 min in a solution of periodic acid 1% (*v/v*) and acetic acid 3% (*v/v*). Afterwards, the gel was washed in distilled water and then in 0.1% (*w/v*) sodium metabisulfite solution (10 mM HCl). The gel was then stained with Schiff’s reagent in the dark for 1 h. Afterwards, the gel was incubated for 1 h in 0.1% (*w/v*) sodium metabisulfite solution (10 mM HCl) and finally washed several times in 0.5% (*w/v*) sodium metabisulfite (10 mM HCl) solution. Peroxidase from horseradish (Sigma-Aldrich, Milan, Italy) was used as a positive control and BSA as a negative control.

### 2.9. Dynamic Light Scattering (DLS)

Dynamic light scattering (DLS) measurements were carried out on a Brookhaven system. The light source was the second harmonic of a diode Nd:YAG laser (Torus laser, mpc3000, Laser Quantum, Cheshire, UK) (λ = 532 nm) and the scattered intensity was detected by an avalanche photodiode detector (BI-APD) The samples were placed in glass tubes and immersed in a thermostatic unit filled with decahydronaphthalene to match the glass refractive index. The autocorrelation functions (ACF) were analyzed through the Laplace inversion according to a CONTIN algorithm [61].

## 3. Results and Discussion

### 3.1. Fermentation Performance

*Sch. japonicus* # UCD2489 showed a low fermentative activity. After five days of alcoholic fermentation, it reached the maximum quantity of CO_2_ produced (~ 4.2 g/100 mL) (data not shown). These results are in agreement with those previously observed with *Sch. japonicus* # UCD2489 during the alcoholic fermentation carried out using the same synthetic grape juice but containing a higher sugar concentration (220 g/L) [51].

### 3.2. Polysaccharides Quantification and Characterization

**Figure 1:**
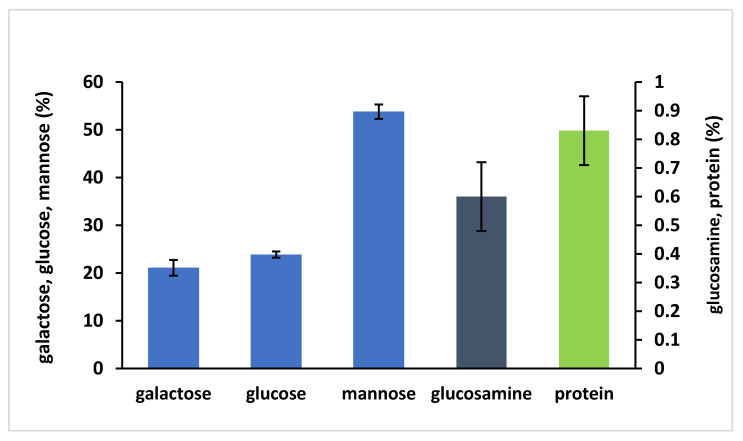
Monosaccharidic composition (expressed as mol %) and proteins content of the freeze-dried polysaccharides (UFS). Error bars represent standard deviation of three analytical replicates, each referred to as experimental duplicates.

After ten days of alcoholic fermentation, the quantity of PSs released by *Sch. japonicus* in the medium was 1.23 ± 0.06 g/L. Figure 1 shows the monosaccharidic composition of the freeze-dried polysaccharide (UFS). The experimental data highlighted a high percentage of mannose (53.8 ± 1.5%) and similar percentages of galactose (21.1 ± 1.7%) and glucose (23.9 ± 0.7%). Instead, the percentages of glucosamine (0.6 ± 0.1%) and proteins (0.8 ± 0.1%) were very low. These results confirm the ability of *Sch. japonicus* yeast to release a high quantity of polysaccharides in the growth medium [51] as compared to 0.05–0.175 g/L usually obtained by *Saccharomyces* yeasts [16,20]. Domizio et al. [51] found a similar composition of monosaccharides and proteins in the PSs released by the same strain of *Sch. japonicus* during the alcoholic fermentation in the same type of synthetic grape juice, but containing Tween 80, ergosterol and a higher sugar concentration. These results are also consistent with the chemical composition of the cell wall of *Schizosaccharomyces* genus, containing galactomannans in the outer layer [62].

**Figure 2:**
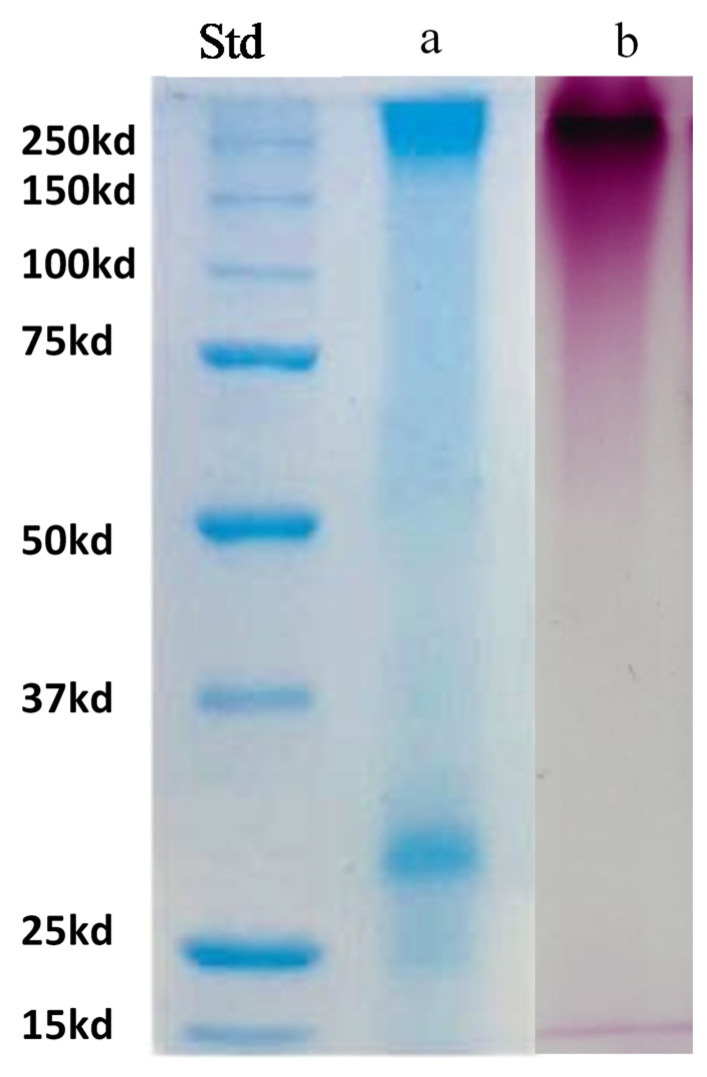
Sodium dodecyl sulfate-polyacrylamide gel electrophoresis (SDS-PAGE) of the freeze-dried polysaccharides (UFS). The glycoproteins were stained on the electrophoretic gel with Bio safe Coomassie (**a**) and with Schiff’s reagent (**b**). Std: Blue precision plus molecular weight standards.

The pattern of the UFS proteins, as analyzed by SDS PAGE, is reported in Figure 2. Total protein profiles were displayed using the Bio-Safe Coomassie G-250 stain (Figure 2a). This analysis revealed a band centered around 32 kDa and bands with molecular-mass greater than 250 kDa. The gel staining with Schiff’s reagent (PAS) (Figure 2b) highlights that the high molecular bands corresponded to glycosylated proteins. Instead, the band around 32 kDa, present in the gel stained with Coomassie, was not evident in the one stained with PAS. In a previous paper, Domizio et al. [51], analyzing the protein profile of the polysaccharides released in the medium by *Sch. japonicus* #UCD2489, showed that the band around 32 kDa corresponded to a glycosylated protein, as demonstrated after gel staining with the Pro-Q Emerald 488 gel stain kit specific for glycoproteins. Considering that Pro-Q Emerald 488 is roughly 8–16-fold more sensitive than the Schiff’s reagent [63], the band around 32 kDa, even if it is not evidenced by PAS (Figure 2b), could be associated to a glycoprotein.

### 3.3. Impact of PSs on Wine Protein Stability

**Figure 3:**
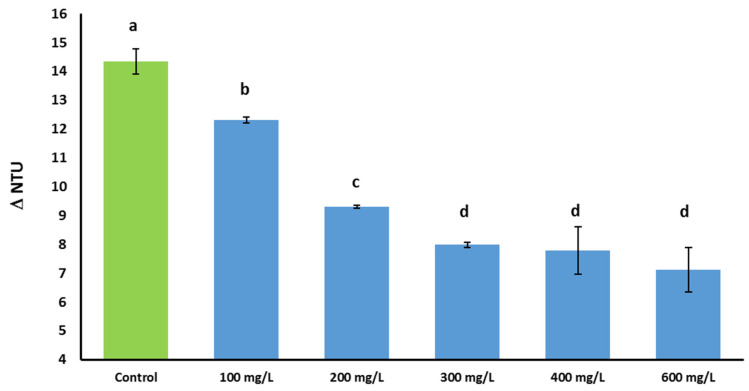
Haziness of Vernaccia wine added with increasing amounts of UFS (from 100 mg/L to 600 mg/L) as obtained by nephelometry after heating treatment. Control: Vernaccia wine without UFS. Error bars represent standard deviation of three independent experiments, each carried out in duplicate. Values displaying different letters (a, b, c, d) are significantly different (One-way ANOVA, Tukey’s post-test, *p* = 0.05).

The impact of increasing concentrations of *Sch. japonicus* polysaccharides (UFS) on heat-induced protein haze formation in the Vernaccia wine was assessed by nephelometry and dynamic light scattering. All the measurements were also performed on a control sample (i.e., an aliquot of wine where UFS was not added). Figure 3 shows the difference in nephelometric turbidity units (ΔNTU) between heated and unheated samples as a function of the different amounts of UFS added. One-way ANOVA and Tukey’s post-test showed significant differences for all samples, except for 300, 400, and 600 mg treatments (*p* = 0.05). The control sample showed a ΔNTU of 14.4 ± 0.3. The visible haziness induced by heating, due to the destabilization/aggregation of proteins naturally present in the wine, decreases as the concentration of added PSs increases, revealing an inverse exponential relationship between the level of haze protection and the concentration of the additive. In particular, the addition of 100 mg/L of UFS resulted in a 14% reduction in haziness compared to untreated wine (control). A further 20% decrease was observed following the addition of an extra 100 mg/L of UFS. Further additions of UFS result in lesser percentage decrease in haziness, reaching a 50% total reduction compared to the control with the addition of 600 mg/L of UFS. A similar relationship between turbidity and concentration of macromolecules with haze protective activity was observed also by other authors [26,29,64]. 

Taking into account that wines are usually considered protein stable when the difference in turbidity between heated and unheated controls are less than 2 ΔNTU [65], these results show that the addition of 300 mg/L of PSs into a wine is able to reduce protein haze to around 50% of the initial value.

**Figure 4:**
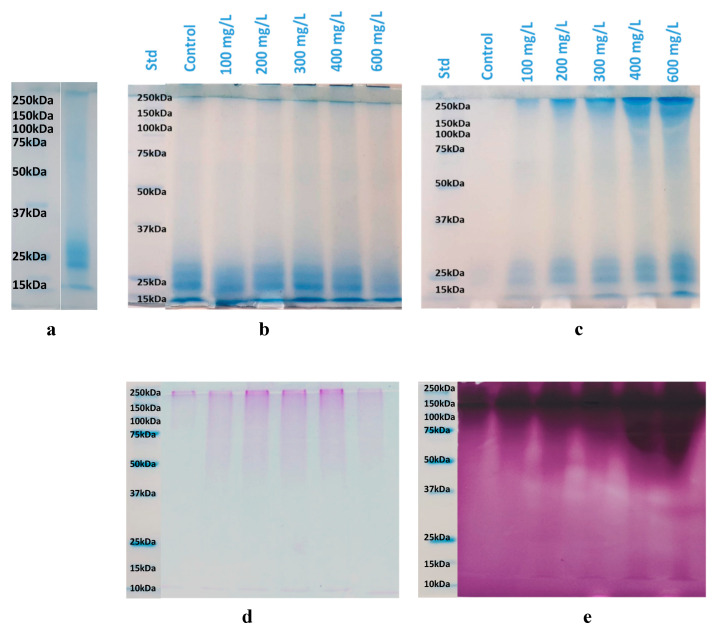
SDS-PAGE electrophoresis of Vernaccia wine proteins before the heat stability test (**a**) and of the two fractions, haze (HF) (**b**–**d**) and supernatant (SF) (**c**–**e**), obtained after centrifugation of the wines added with increasing amount (mg/L) of freeze-dried polysaccharides (UFS) and heat treated. Control: Vernaccia wine with no UFS addition. The proteins were stained on the electrophoretic gel with Bio safe Coomassie (**b,c**) and with Schiff’s reagent (**d,e**). Std: Blue precision plus molecular weight standards.

Figure 4a–d show the electrophoretic profiles of Vernaccia wine (before the heat test) and of the two fractions (HF and SF) obtained after centrifugation of the heat-treated wines added with increasing amount of UFS. The bands around 20–28 kDa, present in the protein profile of Vernaccia wine before the heat test (Figure 4a), could be assigned to grape-derived proteins (PR-proteins), such as chitinase and thaumatin-like proteins (TLPs) [66,67,68]. PR-proteins are considered the main cause of protein haze in white wine, which can occur with extended storage at elevated temperatures [68,69].

SDS-PAGE analysis of the haze fraction (HF) (Figure 4b), showed the presence of proteins bands only within the range of 20–28 kDa. This result emphasizes the heat instability of these proteins, in agreement with their supposed nature as PR proteins. Moreover, as the level of PSs addition increased, a small decrease of the intensities of these bands was observed. In contrast, the added PSs appeared to remain soluble and stable after heating. Indeed, the bands with a molecular-mass greater than 250 kDa, corresponding to those of the added PSs, were clearly lacking in the haze fractions (HF) (Figure 4b) and present in the supernatant fractions (SF) (Figure 4c). Interestingly, an increase in the level of added PSs generates an increase in the intensities of the relevant bands and of those corresponding to 20–28 kDa (Figure 4c). These findings highlight the heat stability of *Sch. japonicus* PSs and their ability to maintain partially the other proteins dispersed, even at elevated temperatures. In contrast, other authors found that, regardless of the addition of invertase or mannoproteins purified from wine, all the grape-derived protein precipitated after heating [26,29]. 

Instead, the band at 32 kDa, ascribable to the UFS polysaccharides (Figure 2a), was not evident in the gels of both fractions (HF and SF) of the heated wine. This could be due to an interaction of denatured proteins with soluble proteins in a co-precipitation mechanism. 

The haze and supernatant fractions analyzed by SDS-PAGE were also visualized using the PAS carbohydrate stain (Figure 4 d,e). While in the gel of the haze fractions no evident bands were present, the supernatant fractions showed a positive response to the carbohydrate stain, with an increase of the band intensities related to the proteins with molecular-mass greater than 250 kDa as the concentrations of UFS increased. These results confirm that no glycoproteins were present in the haze fraction and that the proteins present in UFS fraction were glycosylated. 

Although wine protein solubility likely increased as the concentration of added PSs increased, a plateau in the haziness reduction was observed for UFS doses higher than 300 mg/L. Indeed, similar residual haze values in the heated wine were detected by the nephelometer at UFS concentrations higher than 300 mg/L. Thus, the heat-induced aggregation process of a portion of proteins seems to be independent from the added PSs. On the other hand, other physico-chemical factors might be involved in the protein aggregation process [68]. Among these, pH can strongly affect the stability of wine proteins [70,71] since wine proteins are positively charged at wine pH and electrostatic attractions with negative charged compounds might determine colloidal instability (protein aggregation and precipitation) [71]. Yeast mannoproteins are reported to be neutral or negatively charged and their density was directly correlated to their phosphorus content [72]. Considering that pH can also affect the charge of some polysaccharides [71,72], further studies are still needed to evaluate the charge density profile of the *Schizosaccharomyces*’ galactomannoproteins in the wine to better understand the mechanism at the base of their interference with wine proteins.

**Figure 5:**
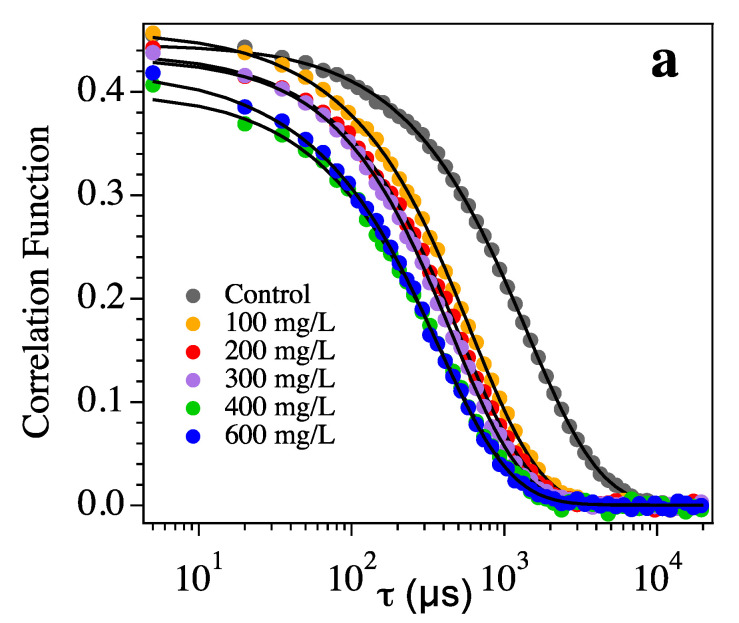

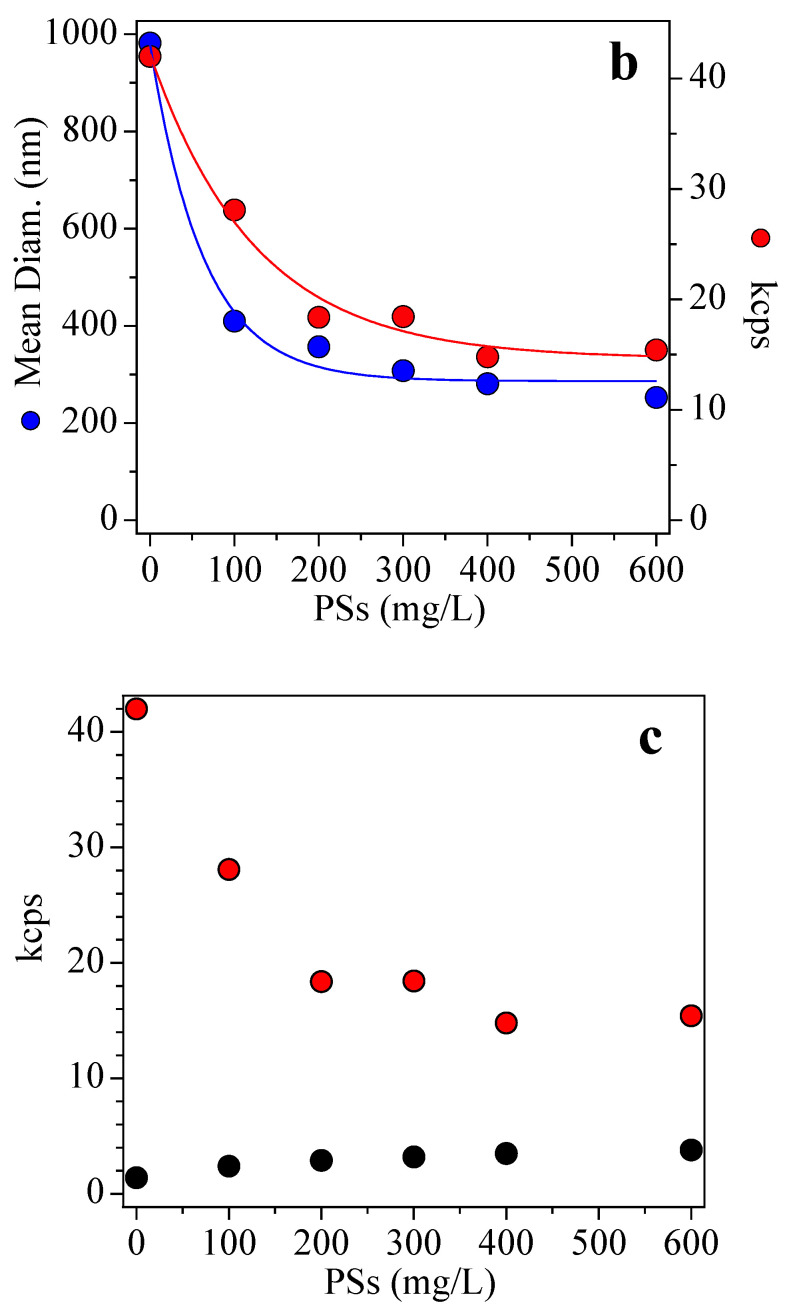
Panel (**a**): correlation functions of the heated samples (dots) along with the CONTIN fit (black lines) vs. the delay time (τ). Panel (**b**): mean diameters (blue) obtained from the fitting of the curves with the CONTIN algorithm together with the number of scattered photons (kilo counts per second (kcps), red). Lines are the best fit according to the exponential function reported in the text. Panel (**c**): comparison between the number of scattered photons in the heated (red) and not heated (black) samples.

Dynamic light scattering (DLS) was also performed on the heat-treated samples of wine to determine the size distribution of the suspended aggregated proteins. DLS measures the Brownian motion of the objects in the sample and relates this to the size of the particles [73]. Figure 5a reports the correlation functions for all the investigated samples, while Figure 5b shows the mean diameters obtained from the fitting of the curves with the CONTIN algorithm together with the number of scattered photons expressed as kilocounts per second (kcps). The comparison between the number of scattered photons in the heated and not-heated samples is shown in Figure 5c. 

The presence of scattering objects in the heated samples can be ascribed to the formation of protein aggregates induced by thermal treatment. Indeed, the comparison between the number of scattered photons in the wine aliquots before and after heating (Figure 5c) confirms the lower number of aggregates in the as-prepared wine samples. Interestingly, the size of the haze particles decreased as the concentration of added polysaccharides increased (Figure 5b). The relationship between the haze particle size and PSs concentration is similar to that found for the turbidity (i.e., exponential function), with the decrease in particle size of the aggregate diminishing to less than 0.3 μm at the highest addition of PSs. As a consequence of this reduction, the haze was barely detectable with the naked eye. Variation of ΔNTU, kcps and mean diameter of the aggregate could be modeled by the function: y(C) = y_0_ + Aexp-(C/C*), where the y_0_ is the baseline of the exponential, A is its amplitude and C* represents the critical concentration of additive able to reduce the destabilization phenomenon by a factor 1/e. C*, resulting from the fits of three curves, is in the range 100–200 mg/L.

These findings are in good agreement with the evidence found by Waters et al. [26] that reported an exponential relationship between haze particle size and the concentration of a crude haze protective factor, with a particle size reduction from 30 μm to 5 μm. 

The release of a high quantity of polysaccharides in the media makes this non-*Saccharomyces* yeast particularly interesting for the industrial production of exogenous polysaccharide preparations that could be then easily purified by ultrafiltration and used for winemaking purposes such as protein stability. Indeed, the possibility to recover high amounts of these compounds directly from the fermentation medium, without using enzymatic treatments or other processes to separate the PSs from the cell wall, could make these yeasts convenient from a production perspective.

## 4. Conclusions

According to our knowledge, this is the first time that *Sch. japonicus* PSs, after being recovered from the growth media by ultrafiltration, have been tested to assess their efficiency on wine protein stability. The results obtained in the present study have clearly shown that these PSs are able to improve wine protein heat stability. In particular, they are able to reduce the protein haze to around half of the initial values. 

The reduction of the haze particles’ size together with the solubility persistence of the heat instable wine proteins following the heat treatment confirm that an addition of these macromolecules into instable wine interferes with the protein aggregation process. To a certain extent, these PSs are able to protect wine from protein haze in a dose-dependent manner. However, further studies are still necessary to characterize and identify the active components of the pool of macromolecules released into the media, to understand their role in the protein haze protection and to evaluate their impact on other chemical, physical, and organoleptic wine features.

## Figures and Tables

**Table 1:** Main analytical parameters of the Vernaccia wine used in the present study.

Chemical Parameters	Values
pH	3.33 ± 0.07
Ethanol % (*v/v*)	12.35 ± 0.14
Residual sugars (g/L)	0.69 ± 0.02
Titratable acidity (as tartaric acid) (g/L)	5.14 ± 0.07
Volatile acidity (as acetic acid) (g/L)	0.23 ± 0.01
Δ * NTU	14.00 ± 0.20

* NTU (Nephelometric Turbidity Units) assessed by the heat test, as reported below (Section 2.7).
